# Healthcare utilization and costs of cardiopulmonary complications following cardiac surgery in the United States

**DOI:** 10.1371/journal.pone.0226750

**Published:** 2019-12-19

**Authors:** Mitali Stevens, Apeksha V. Shenoy, Sibyl H. Munson, Halit O. Yapici, Boye L. A. Gricar, Xuan Zhang, Andrew D. Shaw

**Affiliations:** 1 Global Health Economics & Reimbursement, Edwards Lifesciences, Irvine, California, United States of America; 2 Department of Health Economics and Outcomes Research, Boston Strategic Partners, Inc., Boston, Massachusetts, United States of America; 3 Department of Anesthesiology and Pain Medicine, University of Alberta, Edmonton, Alberta, Canada; Oregon Health & Science University, UNITED STATES

## Abstract

**Purpose:**

This study examined postoperative heart failure (HF) and respiratory failure (RF) complications and related healthcare utilization for one year following cardiac surgery.

**Methods:**

This study identified adult patients undergoing isolated coronary artery bypass graft (CABG) and/or valve procedures from the Cerner Health Facts® database. It included patients experiencing postoperative HF or RF complications. We quantified healthcare utilization using the frequency of inpatient admissions, emergency department (ED) visits with or without hospital admission, and outpatient visits. We then determined direct hospital costs from the determined healthcare utilization. We analyzed trends over time for both HF and RF and evaluated the association between surgery type and HF complication.

**Results:**

Of 10,298 patients with HF complications, 1,714 patients (16.6%) developed persistent HF; of the 10,385 RF patients, 175 (1.7%) developed persistent RF. Healthcare utilization for those with persistent complications over the one-year period following index hospital discharge comprised an average number of the following visit types: Inpatient (1.49 HF; 1.55 RF), Outpatient (2.02, 0.51), ED without hospital admission (0.33, 0.13), ED + Inpatient (0.08, 0.06). Per patient annual costs related to persistent complications of HF and RF were $20,857 and $30,745, respectively. There was a significant association between cardiac surgical type and the incidence of HF, with risk for isolated valve procedures (adjusted OR 2.60; 95% CI: 2.35–2.88) and CABG + valve procedures (adjusted OR 2.38; 95% CI: 2.17–2.61) exceeding risk for isolated CABG procedures.

**Conclusions:**

This study demonstrates that HF and RF complication rates post cardiac surgery are substantial, and complication-related healthcare utilization over the first year following surgery results in significant incremental costs. Given the need for both payers and providers to focus on healthcare cost reduction, this study fills an important gap in quantifying the mid-term economic impact of postoperative cardiac surgical complications.

## Introduction

Cardiac surgery represents a significant burden to the United States (US) healthcare system with over 294,000 coronary artery bypass graft (CABG) and valve replacement/repair procedures performed annually [1, 2]. Persistent impairment of cardiopulmonary function in patients undergoing cardiac surgery typically presents as heart failure (HF) and/or respiratory failure (RF) [3, 4]. While several studies [5–8] examined rates of post-cardiac surgical complications for acute respiratory failure (patients treated largely prior to 2010; [5–7]), studies with a primary objective of examining post-cardiac surgical heart failure are sparse and limited to non-US studies based on data prior to 2010 [9, 10].

US studies examining post-surgical acute RF report rates for prolonged mechanical ventilation, with recent rates of 9.0% (US Commonwealth of Virginia, 2006–2015, [8]), and 14.5% (US Society of Thoracic Surgeons [STS] database, 1993–2007, with rates increasing from 11.9% to 16.5% over this period [1]) reported. Short-term healthcare utilization (and costs associated with HF and RF) during hospitalization is well-documented in the medical literature and available through databases which include the Healthcare Cost and Utilization Project (HCUP) database [11]. The reported mean costs per US hospital stay range from $10,500 to $14,631 for HF [12–15], and $15,987 to $40,744 for RF [8, 16, 17].

The economic impact for treatment of HF and RF complications post cardiac surgery, however, goes beyond short-term index hospitalization. Publications regarding long-term healthcare utilization and costs of treating HF in the US either typically focus on specific populations, such as Veterans’ Affairs beneficiaries with HF (a 98% male, 77% white population), or report aggregate costs [18, 19]. Publications regarding long-term healthcare utilization and costs to treat RF are limited to European studies evaluating specific interventions (i.e. nocturnal noninvasive ventilation) or specific patient subgroups (i.e. patients with chronic obstructive pulmonary disease (COPD); restrictive thoracic disease) [20–24].

There is a gap in the published US healthcare literature surrounding the not-infrequent complications of HF and RF following cardiac surgical index visits. We utilized a large US electronic health records (EHR) database to evaluate the rates of these complications by year and procedure type among adult patients that underwent CABG and/or valve repair/replacement procedures. Healthcare utilization rates and costs for treating these complications over the year following surgery are presented.

## Methods

We conducted a retrospective study using a US EHR database (Cerner Health Facts^®^, Kansas City, MO) which includes Health Insurance Portability and Accountability Act (HIPAA) compliant clinical and administrative data from 720 US hospitals and health systems, including 69 M patients. Western IRB (Puyallup, WA, USA) approved the study protocol and analysis plan in advance of data extraction and analysis.

### Patient population for index visit

We analyzed patients who underwent isolated CABG and/or valve procedures, identified via International Classification of Disease (ICD) 9 and 10 procedure codes (Table A in S1 File), between January 1, 2011 and December 31, 2016. The study included adult patients (≥ 18 years of age) with a minimum 24-hour hospital length-of-stay (LOS ≥ 24 hours) for the surgical index visit. We defined the index visit for each patient as the first of multiple qualifying procedures in the database. We evaluated patients for development of acute complications, either heart failure (HF) and/or respiratory failure (RF), during their surgical index visit using ICD- 9 and 10 codes (Table B and Table C in S1 File, respectively). This study identified newly developed complications by excluding those that were present on admission and/or were the primary reason for admission.

### Patient population for one-year follow-up period

This study included patients who experienced HF or RF complications within the index cardiac surgical visit and with data available in the database over the one-year follow-up period. To determine whether these complications persisted in the period after index visit discharge, we performed the following steps to obtain patient cohorts who required care in the year following their index cardiac surgical procedure.

#### Persistent heart failure cohort

To limit the evaluation to patients with *newly developed* HF during the surgical index visit, the study excluded patients who experienced postoperative acute on chronic HF (Table D in S1 File) within the surgical index visit from the final “persistent HF” cohort. We identified patients with persistent HF as those with at least two visits in the patient record over the follow-up period with a diagnosis for the visit being HF-related (Table E in S1 File).

#### Persistent respiratory failure cohort

The study identified patients with persistent RF as those with at least two visits in the patient record over the one-year follow-up period with a diagnosis for the visit being RF-related (Table F in S1 File). To restrict the final “persistent RF” cohort to patients with RF unrelated to chronic obstructed pulmonary disorder (COPD), we excluded patients with at least two visits within the patient record over the follow-up period with a primary diagnosis of COPD for the visit (Table G in S1 File).

### Healthcare utilization and costs during one-year follow-up

The study quantified healthcare utilization for patients experiencing persistent HF or RF complications during the one-year follow-up period including inpatient stays, emergency department (ED) visits resulting in an inpatient stay, ED visits without a hospital admission, and outpatient visits.

This study obtained costs through US datasets and published US sources. We determined HF- and RF-specific inpatient costs from the US HCUP database (Agency for Healthcare Research and Quality, Rockville, MD [11]). We extracted ED costs from published references identified via searches performed in PubMed, Embase, and Google Scholar databases [19, 25]. We calculated outpatient or physician office visit costs by assigning a Medicare Reimbursement Rate to relevant CPT codes [26]. The study inflated all costs to December 2018 US dollars (USD) using the United States Bureau of Labor and Statistics Consumer Price Index (CPI) inflation calculator [27]. We calculated one-year follow-up costs by multiplying the average annual number of visit types (inpatient, ED without a hospital admission, ED + inpatient, outpatient) by the cost of the visit type. This study did not specifically add a mortality factor to the cost calculation as it is included within the average healthcare utilization values over the year.

### Statistical analyses

The study summarized baseline patient characteristics via counts and percentages for binary or categorical variables and with means and standard deviations for continuous variables. To evaluate trends over time for both postoperative HF and RF, we used the Cochran-Armitage test. We evaluated the association of post-surgical HF, and separately, RF, with isolated CABG, isolated valve, or CABG + valve procedures using a Chi-square test. We used a logistic regression model to quantify the relationship between surgical type and the complication of heart failure, adjusting for patient characteristics (surgery type and year, Charlson index [28], gender, race, admission type [e.g. emergency, urgent, elective]) and hospital characteristics (census region, bed size, urban/rural, and teaching status). We conducted the study analyses using SAS version 9.4 (SAS Institute Inc., Cary, NC, USA).

## Results

### Cardiac surgical index visit

**Fig 1:**
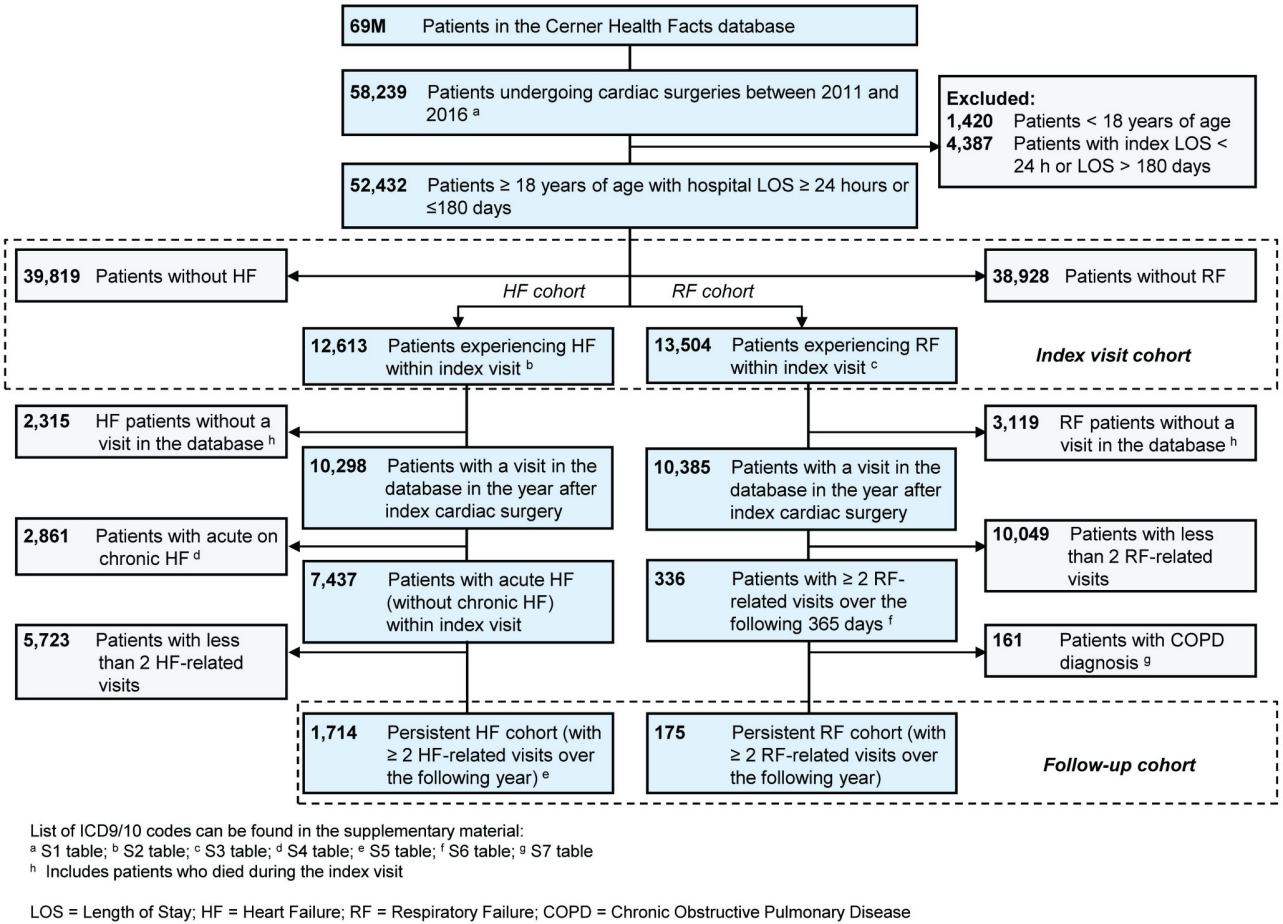
Patient selection and flow diagram.

We identified 52,432 patients who underwent cardiac surgery between January 1, 2011 and December 31, 2016 and fulfilled study inclusion criteria; 12,613 (24.1%) patients who met study criteria experienced heart failure during the index admission while 13,504 (25.8%) experienced respiratory failure (Fig 1; Table 1 demographics).

**Table 1:** Patient and hospital baseline demographics* of the index visit cohort.

	Heart failure			Respiratory failure		
Characteristics	Yes (n = 12,613) n (%)	No (n = 39,819) n (%)	p-value	Yes (n = 13,504) n (%)	No (n = 38,928) n (%)	p-value
**Surgery year**			<0.001			<0.001
2011	1,050 (8.3)	5,691 (14.3)		1,904 (14.1)	4,837 (12.4)	
2012	1,425 (11.3)	5,719 (14.4)		2,002 (14.8)	5,142 (13.2)	
2013	2,708 (21.5)	7,778 (19.5)		2,738 (20.3)	7,748 (19.9)	
2014	2,688 (21.3)	7,628 (19.2)		2,464 (18.2)	7,852 (20.2)	
2015	2,552 (20.2)	6,581 (16.5)		2,319 (17.2)	6,814 (17.5)	
2016	2,190 (17.4)	6,422 (16.1)		2,077 (15.4)	6,535 (16.8)	
**Surgery type**			<0.001			<0.001
CABG	6,561 (52.0)	27,359 (68.7)		7,738 (57.3)	26,182 (67.3)	
Valve	4,144 (32.9)	8,843 (22.2)		3,771 (27.9)	9,216 (23.7)	
CABG + Valve	1,908 (15.1)	3,617 (9.1)		1,995 (14.8)	3,530 (9.1)	
**Charlson Index,** mean (SD)	7.33 (2.60)	5.3 (2.45)	<0.001	6.43 (2.75)	5.57 (2.55)	<0.001
**Gender**			<0.001			<0.001
Female	4,659 (36.9)	11,757 (29.5)		4,678 (34.6)	11,738 (30.2)	
Male	7,919 (62.8)	27,388 (68.8)		8,470 (62.7)	26,837 (68.9)	
Not specified	35 (0.3)	674 (1.7)		356 (2.6)	353 (0.9)	
**Age group**			<0.001			<0.001
18–34	191 (1.5)	590 (1.5)		245 (1.8)	536 (1.4)	
35–49	875 (6.9)	2,944 (7.4)		1,049 (7.8)	2770 (7.1)	
50–64	3,909 (31.0)	13,788 (34.6)		4,426 (32.8)	13,271 (34.1)	
65+	7,638 (60.6)	22,497 (56.5)		7,784 (57.6)	22,351 (57.4)	
**Race**			<0.001			<0.001
Caucasian	9,617 (76.2)	32,659 (82.0)		10,194 (75.5)	32,082 (6.5)	
African American	1,644 (13.0)	3,040 (7.6)		1,597 (11.8)	3,087 (7.9)	
Other	999 (7.9)	2,539 (6.4)		1,013 (7.5)	2,525 (3.2)	
Not specified	353 (2.8)	1581 (4.0)		700 (5.2)	1234 (82.4)	
**Admission type**			<0.001			<0.001
Emergency	5,391 (42.7)	10,756 (27.0)		5,037 (37.3)	11,110 (28.5)	
Urgent	2,147 (17.0)	5,213 (13.1)		2,095 (15.5)	5,265 (13.5)	
Elective	4,675 (37.1)	21,112 (53.0)		5641 (41.8)	20,146 (51.8)	
Other	41 (0.3)	143 (0.4)		705 (5.2)	158 (0.4)	
Not specified	359 (2.8)	2,595 (6.5)		26 (0.2)	2,249 (5.8)	
**Payer**			<0.001			<0.001
Commercial	2,596 (20.6)	10,708 (26.9)		2,946 (21.8)	10,358 (26.6)	
Medicare	6,764 (53.6)	18,319 (13.3)		6,951 (51.5)	18,132 (8.9)	
Medicaid	784 (6.2)	1,862 (46.0)		844 (6.3)	1,802 (13.3)	
Other	1,134 (9.0)	3,645 (9.2)		1,313 (9.7)	3,466 (46.6)	
Not specified	1,335 (10.6)	5,285 (4.7)		1,450 (10.7)	5,170 (4.6)	
**Census region**			<0.001			<0.001
Midwest	1,584 (12.6)	6,084 (15.3)		2,410 (17.8)	5,258 (13.5)	
Northeast	1,859 (14.7)	7,090 (17.8)		2,212 (16.4)	6,737 (17.3)	
South	6,997 (55.5)	21,343 (53.6)		6,816 (50.5)	21,524 (55.3)	
West	2,173 (17.2)	5,302 (13.3)		2,066 (15.3)	5,409 (13.9)	
**Bed size**			<0.001			<0.001
<5	164 (1.3)	513 (1.3)		179 (1.3)	498 (1.3)	
6–99	1,589 (12.6)	5,062 (12.7)		1,137 (8.4)	5,514 (14.2)	
100–199	1,133 (9.0)	3,094 (7.8)		998 (7.4)	3,229 (8.3)	
200–299	2,300 (18.2)	7,461 (18.7)		2,661 (19.7)	7,100 (18.2)	
300–499	2,337 (18.5)	8,533 (21.4)		2,774 (20.5)	8,096 (20.8)	
500+	5,090 (40.4)	15,156 (38.1)		5,755 (42.6)	14,491 (37.2)	
**Teaching**			<0.001			<0.001
Yes	8,473 (67.2)	27,706 (69.6)		9,794 (72.5)	26,385 (67.8)	
No	4,140 (32.8)	12,113 (30.4)		3,710 (27.5)	12,543 (32.2)	
**Urban**			<0.001			<0.001
Yes	10,718 (85.0)	35,333 (88.7)		11,401 (84.4)	34,650 (89.0)	
No	1,895 (15.0)	4,486 (11.3)		2,103 (15.6)	4,278 (11.0)	

CABG = Coronary Artery Bypass Graft

*Baseline data were compared using t tests for continuous variables and chi-square tests for categorical variables

#### Complication trends

**Fig 2:**
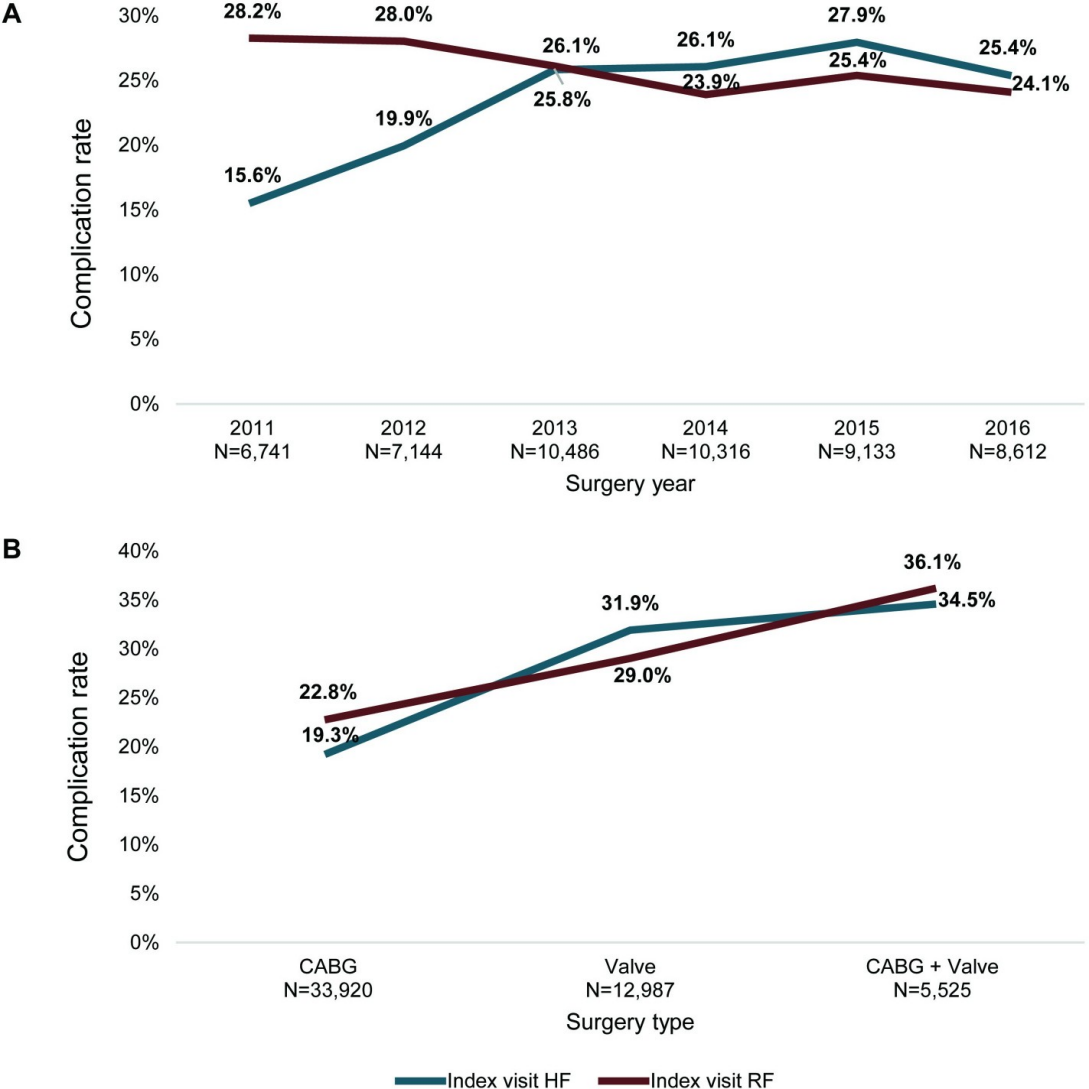
Rates* of heart failure (HF) and respiratory failure (RF) for the index visit by a) surgical year (2011–2016); b) surgical type (CABG, Valve, CABG + Valve). A) There was a significant difference in the rate of HF and RF over time (p<0.0001 for each); B) There was a significant association between surgical type (isolated CABG, isolated valve, or CABG + valve) and complication rates for HF or RF (p<0.001 for each complication). *All rates are expressed as percentages of each category.

Over the study period (2011 to 2016), the rate of HF post cardiac surgery increased (p<0.0001), while conversely, the rate of RF complications decreased over this time period (p<0.0001) (Fig 2A) when evaluated via the Cochran-Armitage trend test.

#### Association between cardiac procedure type and complications

Chi-square tests revealed that there was a significant association between surgical type (isolated CABG, isolated valve, or CABG + valve) and complication rates for HF or RF (p<0.001 for each complication). Patients who underwent CABG + valve procedures had the highest complication rates (34.5%, HF; 36.1%, RF); patients who underwent isolated CABG procedures had the lowest complication rates (19.3%, HF; 22.8%, RF) (Fig 2B).

Given the significant association between a HF complication and cardiac surgical type, and the observed increase in HF complications over time (2011–2016), we performed a logistic regression analysis, adjusting for patient and hospital characteristics (Table 2). Logistic regression modeling revealed that the odds of HF for patients undergoing CABG + valve procedures is 2.38 times the odds for an isolated CABG procedure (Table 2; 95% CI: 2.17–2.61), and the odds of HF for isolated valve procedures is 2.60 times the odds for isolated CABG procedures (Table 2; 95% CI: 2.35–2.88).

**Table 2:** Logistic regression model results for heart failure cohort at index visit (n = 52,432).

	Odds ratio (95% confidence interval)	p-value
**Surgery type**		
CABG + Valve vs CABG	2.38 (2.17–2.61)	< .0001
Valve vs CABG	2.60 (2.35–2.88)	< .0001
**Surgery year**	1.04 (0.98–1.10)	0.2568
**Charlson Index**	1.33 (1.31–1.36)	< .0001
**Gender**		
Female vs Male	1.09 (1.03–1.15)	0.0047
Unknown vs Male	0.37 (0.13–1.08)	0.0675
**Race**		
African American vs Caucasian	1.66 (1.44–1.91)	< .0001
Other vs Caucasian	1.28 (1.15–1.43)	< .0001
Not specified vs Caucasian	1.23 (0.98–1.54)	0.0723
**Admission type**		
Emergency vs Elective	2.66 (2.31–3.05)	< .0001
Urgent vs Elective	2.17 (1.80–2.63)	< .0001
**Census region**		
Midwest vs South	0.93 (0.69–1.26)	0.6406
Northeast vs South	0.69 (0.39–1.23)	0.2043
West vs South	1.26 (0.94–1.70)	0.1239
**Bed size**		
6–99 vs 500+	0.90 (0.57–1.43)	0.6435
100–199 vs 500+	0.87 (0.58–1.32)	0.5111
200–299 vs 500+	0.81 (0.56–1.17)	0.2508
300–499 vs 500+	0.72 (0.53–0.98)	0.0363
**Urban status**		
Rural vs Urban	1.16 (0.86–1.55)	0.3358
**Teaching facility**	0.91 (0.67–1.22)	0.5129

CABG = Coronary Artery Bypass Graft

Results for admission types ‘other’ and ‘unknown’, and bed size <5 were not statistically significant

#### Patient and hospital demographics associated with heart failure complication rates

Logistic regression modeling of patient/hospital demographics and HF complication rates demonstrated significant increased risk for HF complications associated with the following characteristics (adjusted odds ratios [95% CI]): race of African-American or ‘other’ (race identified as not African-American or Caucasian) vs. Caucasian (1.66 [1.44–1.91] and 1.28 [1.15–1.43], respectively), urgent or emergency vs. elective surgery (2.17 [1.80–2.63] and 2.66 [2.31–3.05], respectively), an increased Charlson comorbidity index (1.33 [1.31–1.36]), and female sex (1.09 [1.03–1.15]). The adjusted odds for experiencing a HF complication was significantly reduced for hospitals of bed-size 300–499 vs. 500+ (0.72, 95% CI: 0.53–0.98).

### One-year post surgery

#### Population with persistent heart failure or respiratory failure complications

We identified 10,298 and 10,385 patients who experienced HF or RF complications during the surgical index visit, respectively, and who had EHR data available for the one-year follow-up period post index hospital discharge (Fig 1). After excluding patients with less than two HF-related follow-up visits and acute on chronic HF episodes, of those patients who experienced *any* HF, 1,714 had *persistent* HF over the one-year following index cardiac surgery (16.6%; Fig 1). In similar fashion, after excluding patients with a COPD diagnosis and less than two RF-related follow-up visits, there were 175 patients that experienced *persistent* RF over the one-year following index discharge (1.7%; Fig 1). When considering survivors of the index surgical visit with follow-up data available in the one-year period (N = 42,640), 4.0% had persistent HF (N = 1,714) and 0.4% had persistent RF (N = 175). Patients with persistent complications were largely male (HF: 60.3%, RF: 51.4%), Caucasian (HF: 75.1%, RF: 66.5%), and ≥ 65 years of age (HF: 59.1%, RF: 60.3%) (Table 3).

**Table 3:** Patient and hospital demographics for patients with persistent complications (based on index visit).

	Heart failure (n = 1,714) n (%)	Respiratory failure (n = 175) n (%)
**Surgery year**		
2011	151 (8.8)	15 (8.4)
2012	201 (11.7)	27 (15.1)
2013	361 (21.1)	32 (17.9)
2014	396 (23.1)	29 (16.2)
2015	373 (21.8)	39 (21.8)
2016	232 (13.5)	37 (20.7)
**Surgery type**		
CABG	935 (54.6)	84 (46.9)
Valve	520 (30.3)	54 (30.2)
CABG + Valve	259 (15.1)	41 (22.9)
**Charlson Index,** mean (SD)	7.66 (2.64)	7.7 (2.84)
**Gender**		
Female	681 (39.7)	83 (46.4)
Male	1,033 (60.3)	92 (51.4)
Not specified	-	4 (2.2)
**Age group**		
18–34	20 (1.2)	6 (3.4)
35–49	127 (7.4)	12 (6.7)
50–64	554 (32.3)	53 (29.6)
65+	1,013 (59.1)	108 (60.3)
**Race**		
Caucasian	1,288 (75.1)	119 (66.5)
African American	312 (18.2)	34 (19.0)
Other / Not specified	114 (6.7)	26 (14.5)
**Admission type**		
Emergency	754 (44.0)	69 (38.5)
Urgent	202 (11.8)	28 (15.6)
Elective	737 (43.0)	73 (40.8)
Other / Not specified	21 (1.3)	9 (5.0)
**Payer**		
Commercial	364 (21.2)	23 (12.8)
Medicare	944 (55.1)	107 (59.8)
Medicaid	147 (8.6)	17 (9.5)
Other / Not specified	259 (15.1)	32 (17.9)
**Census region**		
Midwest	320 (18.7)	29 (16.2)
Northeast	221 (12.9)	25 (14.0)
South	914 (53.3)	84 (46.9)
West	259 (15.1)	41 (22.9)
**Bed size**		
<100	205 (11.9)	17 (9.5)
100–199	169 (9.9)	15 (8.4)
200–299	313 (18.3)	38 (21.2)
300–499	334 (19.5)	31 (17.3)
500+	693 (40.4)	78 (43.6)
**Teaching**		
Yes	1,163 (67.9)	125 (69.8)
No	551 (32.1)	54 (30.2)
**Urban**		
Yes	1,455 (84.9)	153 (85.5)
No	259 (15.1)	26 (14.5)

CABG = Coronary Artery Bypass Graft

#### Complication-related healthcare utilization during the year following surgery

**Fig 3:**
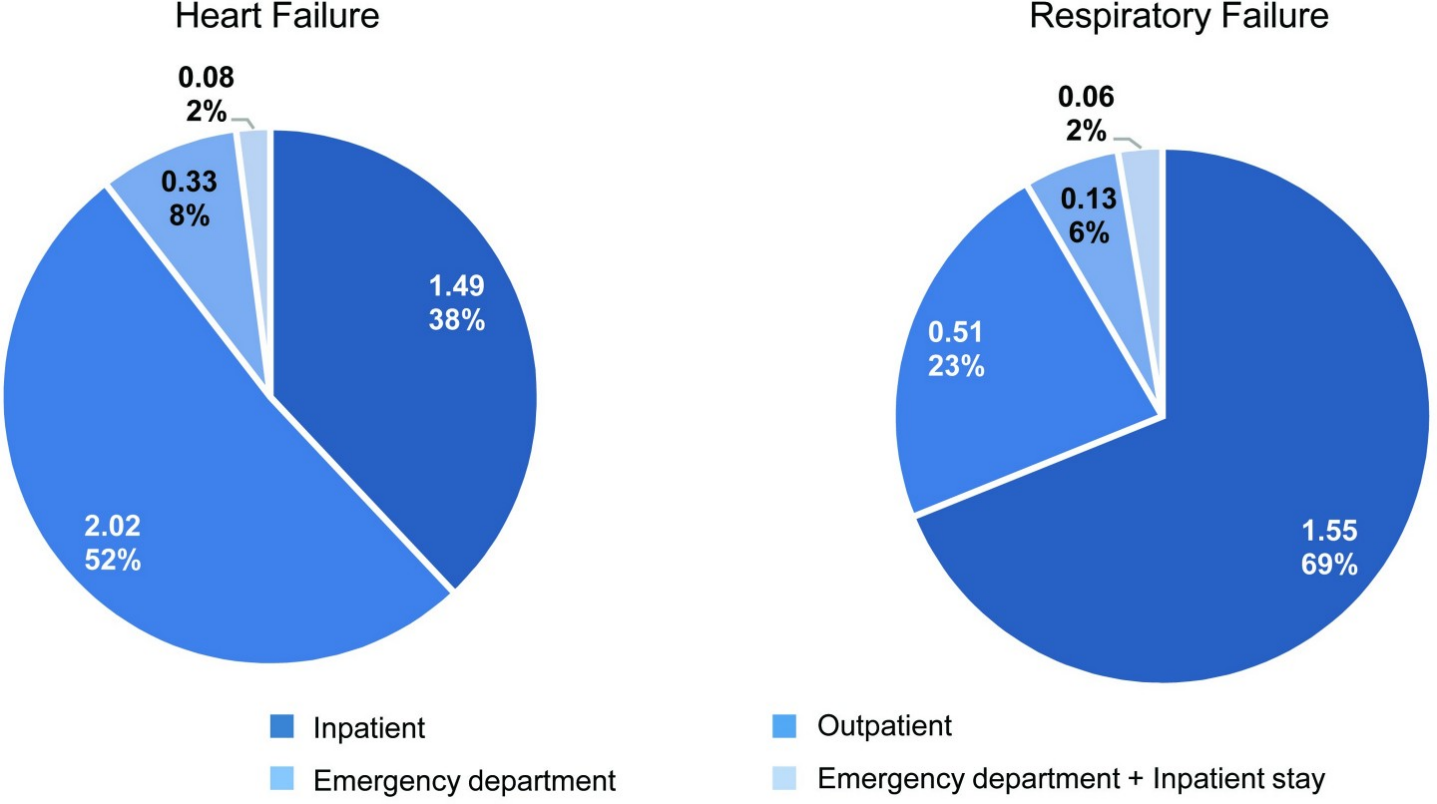
Heart failure- and respiratory failure-related healthcare utilization in the one-year post surgery. Healthcare utilization over the one-year period following index hospital discharge comprised an average number of the following visit types: Inpatient (1.49 HF; 1.55 RF), Outpatient (2.02, 0.51), Emergency Department (0.33, 0.13), Emergency Department + Inpatient (0.08, 0.06).

The study quantified healthcare utilization for patients who experienced newly developed persistent HF and required ongoing treatment over the one-year following surgery (N = 1,714). HF-related healthcare utilization over this period comprised the following average (SD) number of visits: 1.49 (1.53) inpatient stays, 2.02 (2.74) outpatient visits, 0.33 (0.83) ED visits without a hospital admission, and 0.08 (0.46) ED visits resulting in an inpatient stay (Fig 3; Table 4). The resulting average cost for HF complication-related visits among patients with persistent HF was $20,857 per patient over the year following cardiac surgery (Table 4).

**Table 4:** Healthcare utilization by patients requiring treatment for respiratory failure and heart failure complications during the one-year follow-up period.

	Heart failure (HF)			Respiratory failure (RF)		
Visit type ^a^	Number of HF-related visits per patient per year ^b^ (n = 1,714)	Cost per HF-related visit	Total cost of HF visits ^c^	Number of RF-related visits per patient per year ^b^ (n = 175)	Cost per RF-related visit	Total cost of RF visits ^c^
Inpatient	1.49 (1.53)	$12,304^d^	$18,333	1.55 (0.82)	$18,748^d^	$29,059
Outpatient	2.02 (2.74)	$462^e^	$933	0.51 (0.91)	$218^e^	$111
Emergency department	0.33 (0.83)	$1,480^f^	$488	0.13 (0.48)	$2,365^g^	$307
Emergency department + Inpatient ^h^	0.08 (0.46)	$13,784	$1,103	0.06 (0.23)	$21,113	$1,267
**Total**	-	-	$20,857	-	-	$30,745

^a^ Unclassified visits accounted for the following number per patient per year: 0.13 (HF), and (0.09) RF

^b^ Value represented as mean (SD)

^c^ Costs rounded off to the nearest dollar

^d^ [11]

^e^ [26]

^f^ [19]

^g^ [25]

^h^ Emergency department visit resulting in an inpatient stay: Cost inclusive of both ED visit and hospital admission

Similarly, the study quantified healthcare utilization for patients who experienced persistent RF, outside the context of COPD, and required RF complication-related treatment over the year following surgery (N = 175). RF-related healthcare utilization over this period comprised the following average (SD) number of visits: 1.55 (0.82) inpatient stays, 0.51 (0.91) outpatient visits, 0.13 (0.48) ED visits without a hospital admission, and 0.06 (0.23) ED visits resulting in an inpatient stay (Fig 3; Table 4). The resulting average one-year cost of RF-related treatment visits among patients with persistent RF was $30,745 per patient (Table 4).

## Discussion

### Index visit

This study examined over 52,000 patients who underwent CABG, valve, or CABG + valve procedures in the USA between Jan. 1, 2011 and Dec. 31, 2016. Significant associations were observed between cardiac surgery type and the complications of heart failure and respiratory failure (p<0.001 for each complication). Overall, 24% and 26% of patients developed HF and RF complications, respectively. RF in the current study was defined by diagnosis codes for acute RF, procedure codes for mechanical ventilation and endotracheal tube placement, and included select pulmonary insufficiency codes (Table C in S1 File). A published analysis of US cardiac surgery patients with prolonged mechanical ventilation (>24 hrs) between 1993 and 2007 revealed an overall complication rate of 14.5% (STS database) [1]. A more recent study (2006 to 2015) from the US Commonwealth of Virginia, reported a post cardiac surgery prolonged ventilation (>24 hrs) rate of 9.0% [8]. These rates are not directly comparable to the rates in our study as prolonged ventilation > 24 hrs is more restrictive than the RF definition for the current study. Studies which focused on heart failure rates were non-US analyses of patients prior to 2011[9, 29]. Vanky et al. studied a Swedish patient population undergoing aortic valve replacement between 1995 and 2005 and reported HF complication rates of 11.3–11.8% (aortic valve replacement to CABG, respectively) [9]. Since our study placed few restrictions on the study population (e.g., includes all adults, no restrictions on prolonged ventilation), the generalizability of the findings is increased to broader patient population.

Trends analysis revealed that HF complications increased over the study period (p<0.0001), while RF complications decreased over this same period (p<0.0001). This RF trend differs from an earlier analysis examining prolonged mechanical ventilation (1993–2007) in which an increase over this time frame was observed (11.9% to 16.5%) [5]. In the present study, patients who had CABG + valve procedures experienced the highest rates for both HF and RF complications (34.5%, HF; 36.1%, RF), followed by rates for isolated valve procedures, and finally isolated CABG procedures (19.3%, HF; 22.8%, RF). This is not surprising given the fact that patients requiring revascularization in addition to valve surgery are typically sicker, with less well-preserved cardiac function. The size of the effect is important however. Patients undergoing combination surgery were twice as likely to develop cardiopulmonary morbidity as those undergoing isolated CABG (CABG + valve procedures vs. CABG: adjusted OR [95% CI]; 2.38 [2.17–2.61]). The increased incidence of HF complications over time likely reflects the increased underlying severity of cardiac disease present in the modern cardiac surgical population. These rates are significant and would be expected to increase risk for mortality [6, 9, 30, 31], increase treatment costs [12, 13, 31], extend hospital LOS [31], and lengthen time in the intensive care unit.

Logistic regression modeling for heart failure revealed additional factors associated with increased risk for the complication of heart failure including African American or non-Caucasian race, higher Charlson comorbidity index, an urgent or emergency (vs. elective) cardiac procedure, and female gender. Although the clinical significance is unclear, there was a significantly reduced risk of HF for patients admitted to hospitals in the 300–499 bed-size category compared to hospitals with bed-size over 500 (0.72, 95% CI: 0.53–0.98). No other differences in risk were observed for other hospital bed size categories.

### One-year post cardiac surgery

This study found that in patients undergoing cardiac surgery, persistent cardiac dysfunction is more prevalent than persistent RF, with rates of 16.6% and 1.7%, respectively, indicating an almost 10-fold difference. Healthcare utilization for patients with persistent complications in the year following cardiac surgery comprised the following average annual number of visits per patient: Inpatient (HF: 1.49, RF: 1.55), Outpatient (2.02, 0.51), ED without hospital admission (0.33, 0.13), and ED + Inpatient (0.08, 0.06). Visits to an outpatient setting were highest among HF patients, followed closely by inpatient stays. In contrast, RF patients had a higher number of inpatient encounters compared to other visit types. Among all ED visits for HF and RF, 19.5% and 31.6% respectively, resulted in an inpatient stay. When costs per patient per visit type were examined, inpatient costs contributed most to the annual total (HF: $18,333, RF: $29,059). It is possible that more frequent outpatient visits may potentially help reduce inpatient admissions, and thereby costs.

Based on their healthcare utilization, patients who suffered persistent post-surgical HF and RF complications incurred significant costs in the year following index hospital discharge due to need for continuing treatment (HF: $20,857 per patient, RF: $30,745 per patient). If complication rates are minimized in the index visit, this would be expected to not only result in direct, but also indirect cost savings related to loss of productivity, care-giver services, reduced use of community healthcare services, and other related expenses.

### Limitations

This study has several limitations. Although we implemented steps to reduce the risk that complications were erroneously identified, it is probable that coding errors exist which could confound complication identification. The study could detect patient healthcare utilization only if the patient returned to a Cerner facility with a data use agreement. We avoided under-estimation of rates by restricting analysis to patients with established visits spanning the study time period. We identified presence of chronic conditions by examining diagnosis codes during the index visit and in the following year. We did not compare healthcare utilization in the pre versus post hospitalization periods. This study quantified only direct healthcare costs inclusive of care at site of service and did not include indirect costs (e.g. patient transportation, home care, prescriptions, loss of productivity etc.). Unmeasured potential confounding factors may exist for the risk-adjusted model such as surgical duration, treatment choices of cardiac surgeons, cardiac anesthesiologists, and timing surrounding use of vasopressors. However, given the cohort size and number of hospitals, these risks are likely less important.

## Conclusion

This study demonstrates that complications occurring during a cardiac surgical admission have long-term consequences, resulting in additional healthcare utilization and costs to the US healthcare system. Reducing heart failure and respiratory failure complication rates would be expected to improve patient outcomes and result in direct and indirect cost savings. The use of advanced medical technologies and interventions may provide a potential solution; however, comparative clinical and cost effectiveness analyses are needed to determine which products and technologies are most clinically and financially beneficial for preventing post-surgery complications.

## Supporting information

S1 File Supplementary Tables A-G. The supplementary tables file contains a list of all ICD- 9 and 10 codes used in the analysis.
(XLSX)
